# Impacts of Natural Organic Matter and Dissolved Solids on Fluoride Retention of Polyelectrolyte Multilayer-Based Hollow Fiber Nanofiltration Membranes

**DOI:** 10.3390/membranes15040110

**Published:** 2025-04-02

**Authors:** Hussein Abuelgasim, Nada Nasri, Martin Futterlieb, Radhia Souissi, Fouad Souissi, Stefan Panglisch, Ibrahim M. A. ElSherbiny

**Affiliations:** 1 Chair for Mechanical Process Engineering & Water Technology, University of Duisburg-Essen, Lotharstraße 1, 47057 Duisburg, Germany; 7ussien3861@gmail.com (H.A.); martin.futterlieb@uni-due.de (M.F.); 2 Laboratoire des Matériaux Utiles, Institut National de Recherche et d’Analyse Physico-Chimique (INRAP), Technopark of Sidi Thabet, Ariana 2020, Tunisia; nada.nasri66@gmail.com (N.N.); radhia.souissi@inrap.rnrt.tn (R.S.); foued.souissi@fst.utm.tn (F.S.); 3 Department of Geology, Faculty of Sciences of Tunis, University of Tunis El Manar, Tunis 2092, Tunisia; 4 DGMT German Society of Membrane Technology, Geschäftsstelle ZWU, Universitätsstraße 2, 45141 Essen, Germany; 5 IWW Water Centre, Moritzstraße 26, 45476 Mülheim an der Ruhr, Germany

**Keywords:** nanofiltration, polyelectrolyte multilayer-based membranes, groundwater, fluoride removal, sulfate removal

## Abstract

This study examines the effects of natural organic matter (NOM) and dissolved solids on fluoride (F^−^) retention in polyelectrolyte multilayer-based hollow-fiber nanofiltration membranes (dNF40). Lab-scale filtration experiments were conducted under varying operating conditions (initial salt concentration, NOM concentration, permeate flux, crossflow velocity, and recovery rate). dNF40 membranes exhibited F^−^ retention above 70% ± 1.2 in the absence of NOM and competing ions. However, when filtering synthetic model water (SMW) designed to simulate groundwater contaminated with high total dissolved solids (TDSs) and NOM, F^−^ retention decreased to approximately 60% ± 0.7, which was generally attributed to ion competition. Furthermore, despite limited declines in normalized permeability, the addition of NOM to SMW notably deceased F^−^ retention in the steady state to~20% due to fouling effects. The facilitated transport of the divalent cations Ca^2+^ and Mg^2+^ could be observed, as they accumulated in the organic fouling layer. While SO_4_^2−^ retention remained relatively stable, the retention of monovalent anions (NO_3_^−^, Cl^−^, and F^−^) decreased substantially due to drag effects. Na^+^ retention improved slightly to maintain electroneutrality. Feed salinity was shown to significantly affect separation efficiency, with PEC layers undergoing swelling and certain structural changes as the ionic strength increased. During batch filtration experiments at varying recovery rates, the retention of monovalent anions further decreased, with F^−^ retention reducing to just ~10% at a 90% recovery rate. This study provides valuable insights into better understanding and optimizing the performance of PEC-based NF membranes across diverse water treatment scenarios.

## 1. Introduction

Maintaining drinking water quality is a permanent requirement for living beings. The WHO has announced a series of drinking water safety guidelines that include a recommended maximum limit of 1.5 mg·L^−1^ for fluoride (F^−^). However, more than 200 million people in over 20 countries drink water with fluoride concentrations above this guideline limit [1,2]. For instance, in the Gafsa mining basin, Tunisia, phosphate extraction activities have significantly impaired the quality of the local groundwater. The analysis of recently collected samples indicates that the fluoride concentration exceeds the WHO limit, underscoring the need for effective water resource management to protect public health in this region [3]. Mining activities contribute to the release of fluoride into groundwater by disrupting fluoride-containing minerals and facilitating their dissolution. Contaminants such as fluoride and sulfates (SO_4_^2−^) in drinking water can lead to severe health problems [4]. The contamination of local water sources by these pollutants has direct and negative impacts on the health of the regional population exposed to these elements through drinking water [5,6]. Conventional treatment methods such as coagulation–flocculation, activated carbon adsorption, and advanced oxidation processes struggle to effectively remove high fluoride and NOM concentrations. These methods are often ineffective or costly for simultaneously reducing fluoride and NOM levels to acceptable levels [7], necessitating alternative approaches such as adsorption with modified organic/inorganic materials utilizing alumina, clays, calcium-based minerals, synthetic compounds, carbon materials, modified zeolites, ion-exchange resins, and layered double hydroxides and membrane-based techniques like reverse osmosis (RO), nanofiltration (NF), and electrodialysis [8], though scaling up these processes for industrial applications remains a critical challenge [9].

Pressure-driven membrane filtration technologies, RO and NF, have proven effective in removing fluoride and NOM in various hydrogeological contexts. Fluoride transport through NF/RO occurs via diffusion, convection, and electromigration [10,11], while its retention is primarily dictated by size exclusion (i.e., steric hindrance) and electrostatic interactions, including charge repulsion, Donnan exclusion, and dielectric exclusion [12]. In addition to the pore size, charge effects significantly influence fluoride retention, particularly in NF membranes. These factors are largely governed by the water matrix, concentration polarization, and membrane fouling, with the latter two being further impacted by operating conditions such as the permeate flux, recovery (or concentration factor), and crossflow velocity (CFV).

Numerous studies have extensively investigated the application of spiral-wound NF/RO thin-film composite (TFC) membranes for F^−^ removal from various water matrices, examining the impacts of key operational parameters (e.g., the initial feed concentration, permeate flux, recovery, and NOM content) [13,14,15,16]. For instance, M. Tahaikt et al. [17,18] investigated F^−^ removal from underground water spiked with varying F^−^ concentrations of (1.8–22.32 mg·L^−1^) in a pilot plant operated with commercial NF membranes (FilmTec™ NF90, FilmTec™ NF400, FilmTec™ NF270, and TR60). NF90 effectively retained F^−^ but required remineralization, while NF400 performed adequately only at 1.8 mg·L^−1^ F^−^, necessitating a double pass at higher concentrations. Comparatively, TR60 and NF270 achieved satisfactory retention at F^−^ levels ≤ 6.32 mg·L^−1^. The variation in F^−^ removal efficiency was attributed to differences in the membrane properties and water matrix composition. Analogously, FilmTec™ NF270 membranes were investigated for the removal of F^−^ and NOM in natural tropical waters in Tanzania [19,20]. Their application exhibited several challenges, including strong membrane fouling, due to the presence of suspended solids and the complexity of NOM [21]. Shen and Schäfer [19] investigated F^−^ retention for six NF and RO membranes and using 22 water samples collected from Tanzania, with F^−^ concentrations ranging from 2.6 to 240 mg·L^−1^. NF90, BW30, and BW30-LE demonstrated the highest retention (>95%) at 50% recovery, which was explained by the size exclusion mechanism of the hydrated fluoride ions. In a separate study [22], the same authors conducted field trials using local water from northern Tanzania with 18 mg·L^−1^ fluoride and 3 mg·L^−1^ NOM using NF90, NF270, and BW30 membranes. All membranes exhibited high TOC retention due to the size exclusion effect [23]. However, F^−^ retention varied, with NF270 achieving only 80%, while NF90 and BW30 demonstrated superior retention exceeding 97%. Furthermore, Nasr et al. [24] investigated the impact of the water matrix on F^−^ retention from Tunisian groundwater using commercial NF5 and NF9 membranes. Their study examined the influence of the initial F^−^ concentration, as well as the effects of coexisting ions, including Cl^−^, SO_4_^2−^, and Ca^2+^, which are commonly present in groundwater. NF5 and NF9 showed F^−^ retention of 57% and 88%, respectively.

Hollow fiber nanofiltration (HFNF) membranes have garnered significant attention in recent years [25], since HFNF membranes typically does not require intensive pre-treatment. HFNF membranes can be more advantageous when compared with spiral-wound modules, with respect to the spacer-free, backwashable design; ease of cleaning [26]; minimal pretreatment requirements [27]; greater packing density; and larger surface area per module, hence enhancing productivity [25]. HFNF membranes can be fabricated through five primary methods (e.g., directly during phase inversion or via modification with the polymerization, coating, grafting, and self-assembly of polyelectrolytes), each offering distinct structural and functional properties [28]. Self-assembly of polyelectrolyte multilayers (PECs) are a particularly interesting HFNF membrane fabrication method also called layer-by-layer (LbL) coating [29,30,31]. PECs are nanometer-thin films built by alternating layers of polycation and polyanion polymers onto porous membrane supports, e.g., polyether sulfone (PES), that are held together through electrostatic, hydrogen bonding, and hydrophobic interactions [32]. Many studies have explored the applicability of HFNF membranes [33,34,35]. Hadad et al. [36] concluded that sulfonated polyethersulfone HFNF membranes can efficiently remove >90% of Mn^2+^, Fe^2+^, and NOM in domestic groundwater treatment; membrane performance was found to be significantly influenced by the water matrix rather than the NOM concentration. While the charge exclusion mechanism governs Mn^2+^ and Fe^2+^ retention in the absence of hardness, the presence of Ca^2+^ and Mg^2+^ reduces their retention due to charge screening. In contrast, NOM retention remains unaffected by hardness, primarily relying on the size exclusion mechanism.

LbL HFNF membranes have been commercialized in particular by the Dutch company NX Filtration (Enschede, The Netherlands). Their dNF40 module is designed to eliminate microplastics, bacteria, viruses, NOM, dissolved solids (mainly multivalent ions), and micropollutants. As a side effect, the dNF40 may also partially eliminate fluoride if it is present in the raw water to be treated. However, the influence of the water matrix, particularly NOM and dissolved solids, on F^−^ removal remains unclear. It is expected that the removal of monovalent F^−^ is more strongly impacted by water matrix interactions compared to the removal of divalent ions such as Mn^2+^ and Fe^2+^, as previously described in [36].

To address this knowledge gap and gain deeper insights into the mechanisms affecting the passage of F^−^, the present study investigates the effects of NOM- and TDS-containing water matrices, as well as the hydrodynamic conditions, on the F^−^ retention of an LbL HFNF. For reasons of transferability into practice, the commercially available dNF40 membrane was used for all experiments. Filtration experiments were conducted in different operation modes, including steady-state mode (without a concentration factor) and batch mode, using a fully automated lab-scale membrane testing unit. Synthetic model feedwater (SMW) mimicking groundwater contaminated with high TDS and NOM contents (adapted to a real water sample from the mining region of Gafsa, Tunisa) were prepared. Different NOM concentrations in SMW were introduced using a potting soil extract solution (PS) containing humic substances, biopolymers, and hydrophobic organic carbon [37]. The influences of different operating conditions, i.e., the initial feed concentration, CFV, typical inland recovery rates of 70–90%, permeate flux, and NOM concentration, were examined to simulate the impacts of different scenarios on the removal performance.

## 2. Materials and Methods

### 2.1. Membrane and Chemicals

The commercially available dNF40 PEC-based HFNF membrane was used in this study. The specific manufacturing conditions employed are proprietary and confidential. However, based on published data and the filed patents, dNF40 was inferred to possess a negative surface charge [38]. De Grooth et al. [39] investigated the odd–even effect and obtained higher permeability values when terminating with polyanion polystyrene sulfonate (PSS) compared to terminating with 0.5 cationic bilayers (i.e., poly (diallyl dimethylammonium chloride, (PDADMAC)) [40]. The patents show comparable results for salt retention for a PSS terminated membrane with six bilayers of (PDADMAC/PSS) with published experimental data [40,41]. Since NX Filtration intends to employ dNF40 for organic micropollutant (OMP) removal, the integration of a modified sulfone cationic material is probably introduced, since significantly higher removal results for uncharged OMPs were obtained [40]. Therefore, a possible configuration for dNF40 might be (SPDADMAC/PSS)_6_ terminated with anionic PSS.

Lab-scale dNF40 membrane modules were supplied by NX Filtration. The (in–out) modules contained 110 modified polyether sulfone-based fibers with a total membrane active surface area of 0.079 m^2^. Each fiber has an inner diameter of 0.7 mm and a 30 cm length. According to the manufacturer, dNF40 membranes exhibit an MWCO of ~400 Da, a nominal barrier pore diameter of ~0.001 μm, a negative surface zeta-potential at pH 7, and magnesium sulfate (MgSO_4_) retention of >95% (at test conditions: 5.0 mM MgSO_4_, 2.5 bar, 25 °C, CFV of 0.5 m·s^−1^).

For the preparation of SMW, analytical-grade salts of MgSO_4_, sodium sulfate (Na_2_SO_4_), sodium nitrate (NaNO_3_), calcium chloride (CaCl_2_), sodium fluoride (NaF), and sodium chloride (NaCl) were purchased from Carl-Roth GmbH, Karlsruhe, Germany. Potting soil (PS) was purchased from Toom Baumarkt GmbH, Cologne, Germany. Ultrapure-quality water (i.e., conductivity: 0.5 µS·cm^−1^ and TOC: <10 μg·L^−1^; produced by OmniaPure UV-TOC, Stakpure, Niederahr, Germany) was used as the water matrix for the preparation of SMW solutions in this study.

For membrane characterization, glucose was purchased from Carl-Roth GmbH, Karlsruhe, Germany. For membrane cleaning, sodium hypochlorite (NaOCl), sodium hydroxide (NaOH), and citric acid (C_6_H_8_O_7_) were purchased from VWR International, Leuven, Belgium. For membrane storage, sodium metabisulfite (Na_2_S_2_O_5_) was purchased from Carl-Roth GmbH, Karlsruhe, Germany.

### 2.2. Preparation and Characterization of SMW

Prior to SMW preparation, the PS extract was prepared as described in [37,42]. A stock solution was extracted by dispersing 3.75 kg of PS in 15 L of a 1% NaOH solution at temperature of 60 °C overnight, and then the mixture was filtered and stored in a lab refrigerator. A detailed chemical composition of the PS extract was reported in a previous study [37], and the main characteristics of the PS extract are presented in Table 1.

SMW imitating contaminated groundwater in the Gafsa mining region was prepared. The composition and main characteristics of a real groundwater sample as well as the prepared SMW are listed in Table 1. SMW was prepared by dissolving certain amounts of individual salts in ultrapure water. Fixed amounts of PS (measured as TOC) were added to SMW at 5, 15, and 30 mg·L^−1^, and a schematic diagram of SMW preparation is provided in the Supplementary Data in Section S1. It is noteworthy to mention that the fluoride concentration was adjusted in all SMW solutions from 2.5 to 20 mg·L^−1^ to ensure its accurate quantification using the laboratory anion chromatography system by minimizing the overlap of chloride (Cl^−^) and F^−^ peaks. This is important since the real contaminated groundwater samples from the Gafsa region exhibited elevated Cl^−^ concentrations, while the lower detection limit of F^−^ was 0.6 mg·L^−1^.

For every filtration experiment, a 5.0 L of SMW solution was prepared, and the pH was adjusted to 7.5 using 0.5M of NaOH or HCl. The feed solution was kept under constant stirring (~300 rpm) to maintain a well-dispersed solution and prevent the precipitation of the suspended particles. SMW feed samples were analyzed with respect to the TOC concentration (Shimadzu TOC-L, Shimadzu Corporation, Kyoto, Japan), spectral absorption coefficient measured at two wavelengths of UV_254 nm_ and VIS_436 nm_ (Lambda 20, PerkinElmer, Ueberlingen, Germany), turbidity (Nephla, Dr. Lange GmbH & Co. KG, Berlin, Germany), and electrical conductivity (Cond-197i, WTW Instruments, Weilheim, Germany), cf. Table 1. The concentrations of major ions, including anions (SO_4_^2−^, NO_3_^−^, Cl^−^, and F^−^) and cations (Na^+^, Mg^2+^, and Ca^2+^), were measured using ion chromatography systems from Thermo Fisher Scientific, Waltham, MA, USA and Eco IC, Metrohm AG, Herisau, Switzerland, respectively.

### 2.3. Calculation of Permeability, Retention, and Recovery

Permeability was determined using Equation (1):

(1) $$W=\frac{J}{{TMP}_{Net}}$$

where W is the membrane permeability (L·m^−2^·h^−1^·bar^−1^), J is the flux (L·m^−2^·h^−1^), and ${TMP}_{Net}$ is the net transmembrane pressure (bar). The flux was corrected with respect to temperature of 20 °C using Equation (2) [38]:

(2) $$J_{20}={(J}_{T}\cdot e^{\left(-0.0239\cdot (T-20\right)})$$

where $J_{20}$ is the flux corrected at a temperature of 20 °C and $J_{T}$ is the flux measured at the actual temperature (T) measured in (°C). The net transmembrane pressure was determined using Equation (3).

(3) $${TMP}_{Net}=TMP-TM\pi$$

where TMP is the measured transmembrane pressure and TMπ is the calculated transmembrane osmotic pressure (bar) using Equation (4) [43]:

(4) $${TM}_{\pi }=\frac{{EC}_{25{}^{\circ}c}\cdot \left(T+320\right)\;}{925,000}$$

The normalized permeability was determined according to Equation (5):

(5) $$W^{\prime }=\frac{W_{t}}{W_{i}}$$

where $W^{\prime }$ represents the normalized permeability, $W_{t}$ is the membrane permeability measured during the filtration of SMW (with or without NOM) at a certain time (*t*), and $W_{i}$ is the initial membrane permeability expressed as pure water permeability.

The salt retention was calculated via Equation (6):

(6) $$R=1-\left(\frac{C_{P_{t}}}{C_{F_{t}}}\right)$$

where R is the retention, and $C_{F_{t}}$ and $C_{P_{t}}$ (mg·L^−1^) are the measured solute concentrations in the feed and permeate at a given filtration time (t). Permeate samples were collected at certain time intervals and analyzed as described in Section 2.2. It is worth mentioning that if the TOC concentrations in the permeates were below the detection limit of the TOC analyzer (i.e., 0.5 mg·L^−1^), TOC retention values are presented in the form of “>”.

The recovery ∅ was determined based on Equation (7), where ∅ is defined as the ratio of the quantity of the produced permeate $V_{p}$ to the quantity of the feed $V_{f}$.

(7) $$\emptyset =\frac{V_{p}}{V_{f}}$$

The concentration factor $F_{C}$ was determined based on Equation (8), where $F_{C}\;$ is the concentration factor; $C_{C}$ and $C_{F}$ are concentration values in the retentate and feed, respectively; and R is the retention of the membrane (it was fixed in this study to 95% of TOC retention for simplicity):

(8) $$F_{C}=\frac{C_{C}}{C_{F}}=1+\frac{\phi \cdot R}{1-\phi }$$

### 2.4. Lab-Scale Performance Experiments Using NF Membranes

#### 2.4.1. Routine Performance Testing

As received, dNF40 membranes were washed from preservative chemicals by forward flushing of ultrapure water at a CFV of 0.75 m·s^−1^ for 15 min. Thereafter, the membranes were compacted by filtering ultrapure water at five flux values of 5, 10, 15, 20, and 25 L·m^−2^·h^−1^ in consecutive increment and decrement steps; each step lasted for 15 min. During membrane compaction, both the permeate and the retentate were recirculated back to the feed tank.

A routine performance test was conducted via the determination of ultrapure water permeability and single NaF salt retention. The pure water permeability was determined prior to every filtration experiment at a constant flux of 20 L·m^−2^·h^−1^ for 15 min using Equation (1). During single NaF salt filtration experiments, the impacts of the initial feed salt concentration and permeate flux on the F^−^ retention ability were examined. Different NaF feed concentrations of 5.5, 44.0, and 77.0 mg·L^−1^ (equivalent to F^−^ concentration of 2.5, 20.0, and 35.0 mg·L^−1^), as well as different permeate fluxes of 10, 20, and 30 L·m^−2^·h^−1^, were examined for a filtration duration of 4 h at a constant CFV of 0.5 m·s^−1^ and a pH of ~6.5. The filtration experiments were performed under steady-state condition, where both the permeate and retentate streams were circulated back to the feed tank. After every experiment, a forward flush using ultrapure water at CFV of 0.9 m·s^−1^ was applied for 5 min before starting the following experiment. Salt retention was measured every 2 h (permeate samples were collected for 5 min) and the average retention was then calculated, cf. Section 2.3. The solute concentrations in the feed $(C_{F_{t}}$) and permeate $(C_{P_{t}}$) were measured at a given filtration time (t) using an electrical conductivity meter.

Additionally, the single salt retention experiment using an initial NaF feed concentration of 44 mg·L^−1^, permeate flux of 30 L·m^−2^·h^−1^, CFV of 0.5 m·s^−1^, and pH of ~6.5 was repeated with an extended filtration duration of 16 h to evaluate the effects of a prolonged filtration time on the membrane performance and separation efficiency. Salt retention was also measured at 2 h intervals, as previously described.

#### 2.4.2. Studying the Effect of Feed Salinity on the Separation Efficiency of dNF40 Membranes

Prior to mini-plant filtration experiments using SMW, a series of filtration experiments were performed to assess the separation efficiency of the dNF40 membrane under high-salinity feed conditions. For this purpose, model feed solutions comprising glucose and NaCl were prepared. Glucose, as a model neutrally charged compound with a molecular weight of 180 g·mol^−1^, was selected to measure the separation efficiency (via a size-exclusion mechanism) for the PEC, while glucose does not contribute to the ionic strength of the solution. This allows the organic retention behavior of the membrane to be monitored without the interference of ionic interactions.

A glucose model solution with a constant initial concentration of 200 mg·L^−1^ was employed, and the retention was determined through a TOC analysis of feed and permeate samples. The ionic strength of the feed solution was varied using NaCl concentrations of 0, 0.6, 2.0, 4.0, 10.0, and 29.0 g·L^−1^. The filtration experiments were performed at a constant permeate flux of~13 L·m^−2^·h^−1^, CFV of 0.5 m·s^−1^, and pH of ~6.5 for 2 h.

#### 2.4.3. Mini-Plant Filtration Tests Using SMW with NOM at Different Initial Concentrations Employing a Constant Crossflow Velocity

Mini-plant filtration experiments were performed at a constant permeate flux of 20 L·m^−2^·h^−1^, CFV of 0.5 m·s^−1^ for 16 h, and recovery value of ~3% (since both the permeate and retentate were circulated back to the feed tank). This was equivalent to a very low $F_{C}$ value of ~1.02, which can be neglected. Model feed solutions comprising SMW with and without NOM were employed, as described in Section 2.2 and Table 1. After the filtration step, the membrane cleaning protocol was started by mechanical forward flushing of ultrapure water at a CFV of 0.9 m∙s^−1^ for 90 s; this was repeated two times. Thereafter, a two-step chemical cleaning procedure was performed. A mixture of NaOCl and NaOH with 200 mg·L^−1^ Cl_2_ at pH 10 was forward flushed at a CFV of 0.5 m∙s^−1^ for 30 min. The hypochlorite mixture was then washed out via forward flushing of ultrapure water at 0.9 m∙s^−1^ for 5 min. The second chemical cleaning step was undertaken by forward flushing of 5000 mg·L^−1^ citric acid at pH 2.5 followed by ultrapure water flushing. At last, mechanical forward flushing and the pure water permeability measurement were performed three times. In all experiments, the initial pure water permeability of dNF40 membranes was able to be recovered up to >96%, and a quantitative analysis of cleaning efficiency is provided in the Supplementary Data in Section S2. All filtration experiments were repeated at least twice to check the results’ reproducibility. The performance of dNF40 membranes was expressed in terms of normalized permeability and retention. For the membrane retention measurement, feed and permeate samples were collected after 2, 4, 8, and 16 h of filtration. Each sample was collected for 5 min and analyzed with respect to the organic and salt contents, and then the respective retention was calculated using Equation (6).

#### 2.4.4. Mini-Plant Filtration Tests Using SMW with NOM at a Constant Initial Concentration Employing Different Crossflow Velocities

Simulating possible harsh fouling scenarios, lab-scale filtration experiments were conducted using SMW with and without NOM (with a constant initial TOC concentration of 30 mg·L^−1^) using different CFVs of 0.25, 0.50, and 0.75 m·s^−1^, while the other operation parameters and membrane cleaning procedure were employed as described in Section 2.4.3. Different CFVs can influence the concentration polarization phenomenon as well as fouling layer formation inside the lumen. The selection of the CFV in technical applications is primarily based on minimizing energy consumption while ensuring effective fouling control and maintaining separation performance. For typical industrial applications of spiral-wound NF membranes, a CFV of 0.2 m·s^−1^ is normally applied [44], while for the dNF40 membrane, the manufacturer recommends a CFV of 0.2–0.6 m·s^−1^ for full-scale applications based on the membrane length [45]. However, in some lab-scale studies using the dNF40 membrane, a CFV > 0.6 m·s^−1^ was applied to sufficiently reduce concentration polarization [41]. For instance, Jährig et al. [46] observed that increasing the CFV from 0.5 to 1.0 m·s^−1^ (at a constant flux and recovery rate) led to an almost 200% rise in specific energy consumption, while salt retention remained similar. In contrast, reducing the velocity to 0.2 m·s^−1^ lowered energy consumption by approximately 39% compared to 0.5 m·s^−1^ but also decreased salt retention by ~16%.

#### 2.4.5. Mini-Plant Filtration Tests Using SMW with NOM at a Constant Initial Concentration Employing Constant Crossflow Velocity at Different Recoveries

Further filtration experiments were conducted to examine the performance of dNF40 membranes by increasing the concentration in the feed–retentate channel up to typical inland recoveries values of 70, 80, and 90%. SMW with and without NOM with an initial TOC concentration of 5 mg·L^−1^ were employed. The filtration experiments were performed at constant permeate flux of 20 L·m^−2^·h^−1^, CFV of 0.5 m·s^−1^, and pH of ~7.5 for a 2 h filtration duration in batch mode, where the retentate was recirculated back to the feed tank, while the permeate was discharged and collected in a separate tank. The membrane separation performance was measured with respect to the original feed concentration; permeate samples were collected at the end of the filtration duration of 2 h. The recovery rates are of particular interest due to their potential impacts on water reuse efficiency and concentrate management. Notably, Verliefde et al. [44] derives and validates a transport model for the rejection of 21 pharmaceuticals and 6 pesticides, which was assessed in a pilot plant at feed water recoveries of 75, 82.5, and 90%. Analogously, Jährig et al. [46] investigated different recovery rates of 50, 75, and 85% using an HFNF membrane manufactured by Pentair X-Flow BV.

## 3. Results and Discussion

### 3.1. Routine Membrane Performance Testing

**Figure 1:**
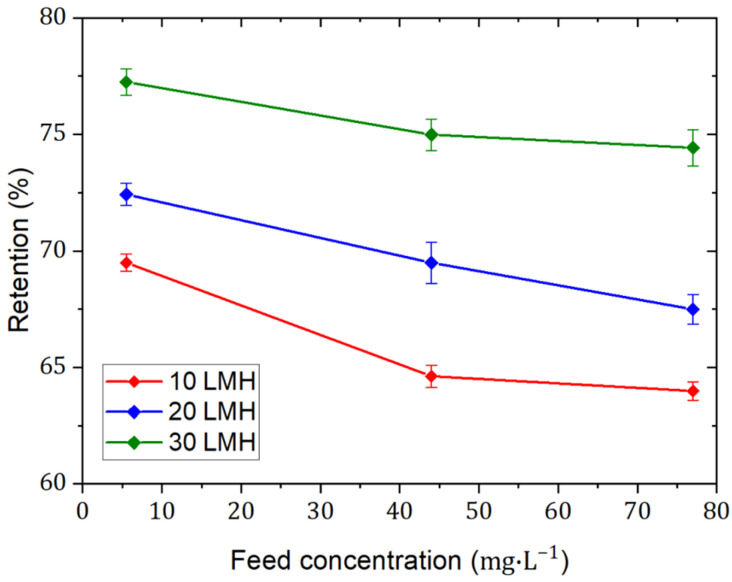
NaF retention measured as electrical conductivity via dNF40 at permeate fluxes of 10, 20, and 30 L·m^−2^·h^−1^ and different NaF feed concentrations of 5.5, 44.0, and 77.0 mg·L^−1^ at a crossflow velocity of 0.5 m·s^−1^, pH of 6.5, and filtration duration of 4 h. Error bars represent the maximum and minimum values.

The results of the single salt filtration experiments (depicted in Figure 1) revealed that NaF salt retention, measured as electrical conductivity, is clearly influenced by both the initial NaF concentration and membrane flux. Specifically, at the same permeate flux of 10 LMH, NaF salt retention decreased from ~70% to ~63% as the initial feed salt concentration increased from 5.5 to 77.0 mg·L^−1^ (equivalent to an F^−^ concentration of 2.5 to 35.0 mg·L^−1^), which is due to a possible weakening of the electrostatic retention or Donnan effect due to an increasing shielding of the membrane charges by counterions. This is a well-known effect of NF membranes [47]. The charge screening effect is, however, counteracted with an increase in the membrane charge due to co-ion adsorption in or on the membrane [10]. However, NaF salt retention increased with increasing membrane flux. For instance, at varying permeate fluxes from 10 LMH to 30 LMH and using an initial feed salt concentration of 44 mg·L^−1^ (equivalent to an F^−^ concentration of 20 mg·L^−1^), NaF salt retention increased from~65% to ~75%, indicating that salt flux and water flux through the membrane are subject to the transport mechanisms of diffusion, convection, and electromigration to varying degrees. These can be modeled using the extended Nernst–Planck equation and are well described in the literature [10,47]. A comparable F^−^ retention trend was reported by Ramdani et al. [48]. The NF270 membrane was found to have F^−^ retention of 70% and 40% during the filtration of 42 and 420 mg·L^−1^ NaF (equivalent to F^−^ concentrations of 19 and 190 mg·L^−1^), respectively, at a pH of 6.5 and a permeate flux of 120 L·m^−2^·h^−1^.

The retention performance by dNF40 membranes can be understood based on the pore-flow model, where steric hinderance and charge effects (such as Donnan and dielectric exclusions) are the main separation mechanisms [49]. Steric hindrance is attributed to the energy barrier imposed on solutes by their size and shape during the partitioning from bulk water into the confined environment in the NF membrane pores [50]. Dielectric exclusion is caused by the interactions of ions with the bound electric charges induced at interfaces between media of different dielectric constants, such as the membrane material and the aqueous solution. The lower dielectric constant inside the membrane pores increases the electrostatic free energy of ions, making it energetically unfavorable for them to enter the pores [51]. Donnan exclusion can be described as preventing the passage of the like-charged ions (co-ions) with respect to the fixed charged groups on the membrane but allowing adsorption and transportation of the counterions [52]. However, the retention of counterions is dictated by the electroneutrality requirements in the feed and permeate solutions. For the single NaF salt retention, it is assumed that at high initial Na^+^ concentrations in the feed, the immobilized negative charges of the membrane (i.e., the polyelectrolyte multilayers), particularly within the pores, are more strongly shielded, reducing the repulsion of the negatively charged F^−^ ions. This suggests a concentration-dependent interaction between Na^+^ ions and the membrane material, which modulates the overall retention of F^−^ ions [53]. The decline in F^−^ retention was aligned and more pronounced from 2.5 to 44.0 mg·L^−^^1^ due to an eightfold concentration increase, whereas the smaller twofold increase from 44.0 to 77.0 mg·L^−^^1^ had a less significant effect.

Interestingly, the improvement of salt retention with increasing membrane flux, as also well-known for denser TFC membranes, indicates that the fluxes of water and charged solutes through the dNF40 membranes are at least partially decoupled. By increasing the TMP to increase the water flux, the flux of charged solutes remained relatively unaffected or increased only slightly due to the charge effects.

Furthermore, the results implied that the employed CFV during the salt retention experiments was sufficient to mitigate the concentration polarization effect on the osmotic pressure. This was evidenced by the near-linear increase in the salt retention with increasing permeate flux, without reaching a plateau that might be expected at suboptimal CFV-to-membrane flux ratios [45]. This also indicates that the CFV was well-matched to the permeate flux ranges tested, preventing concentration polarization-related effects on salt retention.

**Figure 2:**
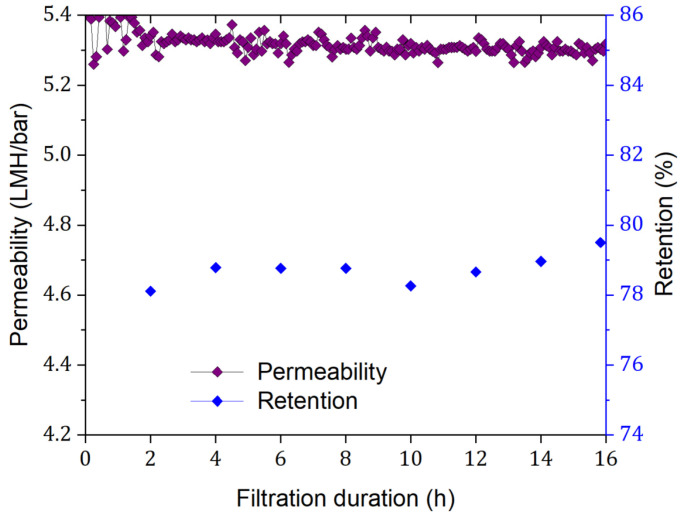
Membrane permeability and NaF retention (measured as electrical conductivity) for dNF40, measured at a permeate flux of 30 L·m^−2^·h^−1^, NaF feed concentration of 44.0 mg·L^−1^, a crossflow velocity of 0.5 m·s^−1^, pH of 6.5, and a prolonged filtration duration of 16 h.

Figure 2 shows the permeability curves for dNF40 during the filtration experiments of the NaF single salt with a feed concentration of 44.0 mg·L^−1^ (i.e., an F^−^ concentration of 20 mg·L^−1^) for a filtration duration of 16 h at permeate flux of 30 L·m^−2^·h^−1^. Throughout the filtration process, no significant decline in the membrane permeability was observed, with a minimal reduction of <1.8% that could rather be attributed to a slight compaction of the membrane multilayers.

### 3.2. The Effect of Feed Salinity on the Separation Efficiency of dNF40 Membranes

Prior to mini-plant filtration experiments with SMW, additional routine membranes testing was conducted to investigate the effects of feed salinity (expressed as ionic strength) on the separation efficiency of the PEC. It is well-known in the literature that increasing the ionic strength in the feed water can influence the detachment, swelling, and roughness of the PEC, and, hence, the separation performance of the NF membranes [54,55,56]. The influences of ionic strength on the separation efficiency of polyelectrolyte multilayer-based membranes were reported to occur through certain mechanisms, including charge screening and polymer swelling [39,57]. Analogously, Luo et al. [58] studied the influence of highly concentrated NaCl salt solutions on the retention of organic solutes by NF polymeric membranes using concentrated glucose and commercial NF membranes (NF270, NF-, Desal-5 DL, and Nanomax50). The results indicated that increasing the salt concentration reduced the solute-to-pore size ratio and increased the membrane thickness-to-porosity ratio, suggesting salt-induced pore expansion, solute size reduction, and membrane swelling. Electrostatic screening at high salt concentrations led to accurate predictions for charged solute retention. The proposed mechanisms include membrane swelling, hydration layer thinning, and particle collisions.

**Figure 3:**
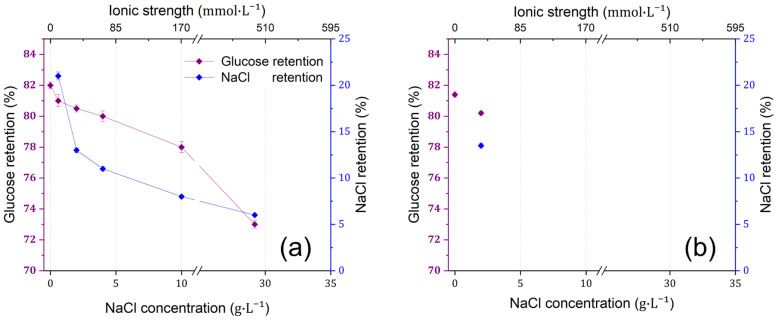
(**a**) Glucose retention measured by a TOC analysis of a 200 mg·L^−1^ solution and NaCl retention measured as electrical conductivity via dNF40 at a permeate flux of~13 L·m^−2^·h^−1^ with different NaCl concentrations of 0, 0.6, 2.0, 4.0, 10.0, and 29.0 g·L^−1^ at a crossflow velocity of 0.5 m·s^−1^, pH of 6.5, and filtration duration of 2 h. (**b**) Investigation of PEC layer recoverability using glucose retention measured at NaCl concentrations of 0 and 2.0 g·L^−1^ under the same operating conditions after the high ionic strength trials in (**a**). Error bars represent the maximum and minimum values.

As depicted in Figure 3a, glucose retention by dNF40 membranes was initially measured at 82% in the absence of NaCl, while it was found to decrease with increasing NaCl concentrations. At a NaCl concentration of 10 g·L^−1^, glucose retention dropped to 78% and further declined significantly to 72% at NaCl concentration of 29 g·L^−1^.

The decrease in glucose (neutral molecule) retention with increasing feed salinity (i.e., NaCl concentration in the feed) is mainly interpreted by swelling and other reversible structural changes in the PEC because of the increase in ionic strength [59]. These mechanistic effects and their influences on glucose retention are substantially dependent on the NaCl concentration in the feed. For instance, glucose retention showed a slight decrease of 2% at NaCl concentrations ≤ 4 g·L^−^^1^, while a decrease of ~10% was measured when the NaCl concentration increased to 29 g·L^−^^1^. Comparable results were reported by Rutten et al. [53] using the dNF40 membrane for the filtration of diethylene glycol, a neutrally charged model compound with a molecular weight of 106 g·mol^−^^1^, at a permeate flux of ~13 L·m^−2^·h^−1^. The initial retention of diethylene glycol in the absence of NaCl was 28%, which decreased slightly to 26% at an NaCl concentration of 3.5 g/L. Both the osmotic pressure and doping mechanism were reported to explain the structural changes in PEC in saline solutions [59]. Depending on the salt concentration, these structural changes can range from reversible (simple) swelling in the polyelectrolyte multilayers (as indicated by a slight decrease in glucose retention at NaCl concentrations ≤ 4 g·L^−^^1^) and pore widening (as indicated by an ~10% decrease in glucose retention at higher NaCl concentrations) to irreversible structural changes (e.g., local polymer chain folding and decreased physical cross-linking) for extremely high salt concentrations (>1 M). These performance results are consistent with reported performance stability results of PEC-based membranes [60,61,62]. Further results observed in Figure 3b revealed that glucose retention measured in absence of NaCl and at concentration of 2 g·L^−^^1^ after the high ionic strength trials in (a) was significantly reversible, indicating that the PEC layer recovers from the influence of the ionic strength.

Moreover, the significant decrease in NaCl retention (from 22% to <6%) can be rather attributed to PEC doping and charge screening mechanisms, where abundant Na^+^ and Cl^−^ ions and water molecules can diffuse into PEC with high saline concentrations and neutralize the charges of the polyelectrolyte multilayers [31,57]. These effects can result in the deactivation of the Donnan exclusion mechanism and a loose PEC structure (due to swelling), which can both lead to lower salt retention.

These findings may indicate that a possible reduction in F^−^ retention might be observed when filtering SMW through the membrane in comparison to the measured retention values in case of single salt experiments. The SMW solution exhibited an ionic strength of ~133 mmol·L^−1^ and electrical conductivity of ~9 mS·cm^−1^, closely approximating the ionic strength of 8 g·L^−1^ of an NaCl solution that has electrical conductivity of 17 mS·cm^−1^. Therefore, applying different recovery values (see Section 3.5) will increase the ionic strength of the feed solution and potentially further decrease the overall membrane retention.

### 3.3. Mini-Plant Filtration Tests Using SMW with NOM at Different Concentrations Employing a Constant Crossflow Velocity

**Figure 4:**
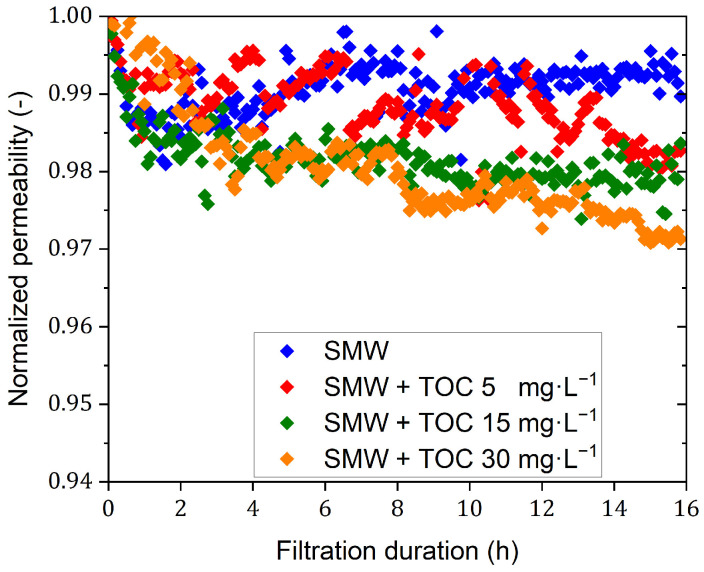
Normalized permeability of SMW filtration using dNF40 with different concentrations of TOC of 5, 15, and 30 mg·L^−1^, a crossflow velocity of 0.5 m·s^−1^, a permeate flux of 20 L·m^−2^·h^−1^, and pH 7.5.

These tests were aimed at investigating the influences of NOM (and membrane fouling) on the separation performance of the dNF40 membranes. The normalized permeability curves are shown in Figure 4. The dNF40 membrane exhibited more-or-less limited normalized permeability declines during the filtration tests with SMW containing different NOM concentrations. The overall normalized permeability decline ratios were <1.0%, 1.5%, 2.0%, and 3.0% for SMW without and with NOM of a TOC of 5, 15, and 30 mg·L^−1^, respectively. These results may imply that increasing the NOM concentration in SMW has no significant influence on fouling layer formation during a 16 h filtration duration. This should be attributed to the high crossflow effect that was able to prevent to some extent, or at least delay, the excessive deposition of organic foulants on the membrane at high NOM concentrations.

However, a general trend can be still noticed that the permeability declines slightly with an increasing NOM dosage. Analogous performance was reported during the filtration of a humic acid solution [20]; only a 3% total reduction in normalized permeability was observed when the humic acid dosage was increased from 5 to 30 mg·L^−1^.

**Figure 5:**
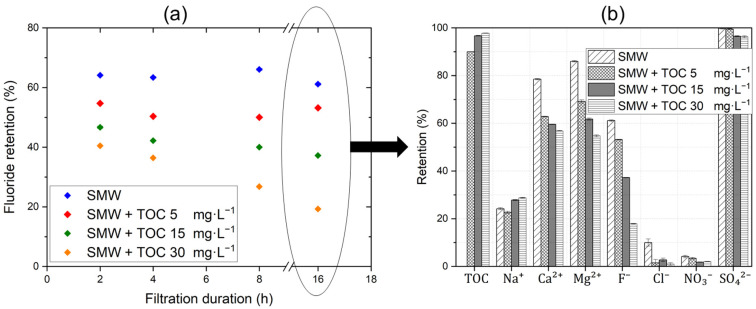
(**a**) Fluoride retention, (**b**) TOC retention, and detailed ion retentions measured during the filtration experiment of SMW containing fluoride with an initial concentration of 20 mg·L^−1^ alone or with the addition of different concentrations of TOC at 5, 15, and 30 mg·L^−1^, a crossflow velocity of 0.5 m·s^−1^, permeate flux of 20 L·m^−2^·h^−1^, and pH 7.5 for 16 h.

Furthermore, the F^−^ retention by dNF40 membranes during the filtration of SMW with and without NOM is plotted in Figure 5. A detailed description of the respective retention values observed during different filtration durations is provided in the Supplementary Data in Section S3. The results show a significant influence of NOM in the feed water on F^−^ retention. During the filtration of SMW without NOM, F^−^ retention remains almost stable in the range of 60–63% throughout the 16 h filtration period that is slightly lower in comparison to the filtration of the single NaF salt solution. This is likely due to the increased ionic interactions and charge screening effects (explained in Section 3.2) that influenced the membrane separation efficiency. However, with increasing NOM concentrations, F^−^ retention decreases significantly until it reaches 20% at a TOC concentration of 30 mg·L^−1^. Nasr et al. [24] concluded that F^−^ retention in NF5 and NF9 membranes is influenced by charge shielding and Donnan equilibrium effects, where Cl^−^ neutralizes the membrane charge, SO_4_^2−^ promotes F^−^ permeation to balance Na^+^, and Ca^2+^ intensifies charge shielding, reducing electrostatic repulsion and weakening membrane–anion interactions. In the study by Owusu-Agyeman et al. [20], the F^−^ retention by NF270 membrane was found to initially increase after humic acid dosing, while it was then decreased by increasing the humic acid dosing. The authors interpreted the first increase in F^−^ retention by the repulsion between the negatively charged F^−^ ions and the fixed negative charges on the membrane; these effects were also enhanced by the initial high permeate flux since the filtration experiments were conducted at a constant pressure. Meanwhile, the decreased membrane flux with increasing humic acid dosing (because of membrane fouling) resulted in a significant decrease in F^−^ ion retention, possibly masking the effects of NOM on concentration polarization effects.

Here, in constant flux filtration experiments using SMW (containing several mono- and multivalent ions), the F^−^ retention decreased directly from the first sampling (i.e., after 2 h), as shown in Figure 5. This effect was promoted with increasing NOM concentrations and at prolonged filtration durations. This significant decline in F^−^ retention can be attributed to the excessively precipitated NOM fouling layer on the membrane. This organic fouling was not completely removed by the crossflow effect, as indicated by the normalized permeability curves in Figure 4. Additionally, the retained divalent cations (Ca^2+^ and Mg^2+^) accumulated within this layer (i.e., lowered back-diffusion) due to their strong affinity for negatively charged NOM substances, as reported in the literature [63], which could be also responsible for the exacerbation of this fouling layer (via NOM-Ca^2+^ complexation) at such high CFVs along the time of experiment. This kind of cake-enhanced concentration polarization effect with respect to the divalent cations and NOM substances was also reported for the NF90 membrane [64].

**Figure 6:**
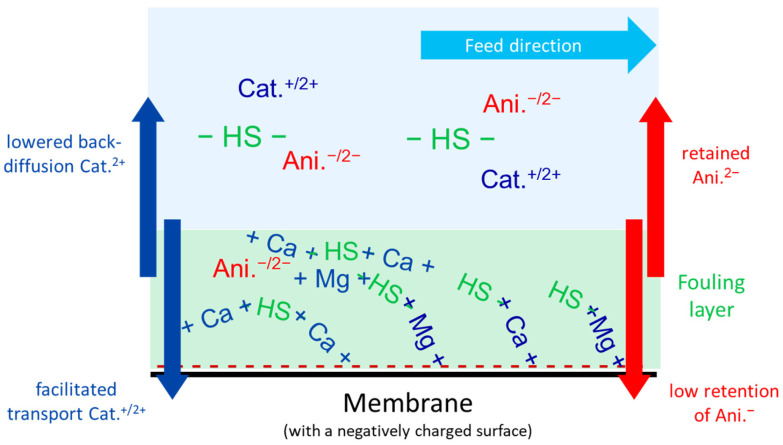
Schematic of ion transport in polyelectrolyte membranes during the filtration of SMW solution with the addition of NOM. The abbreviations used in the graphs are defined as follows: Cat.^+/2+^ for mono/divalent cations, Ani.^−/2−^ for mono/divalent anions, +Ca+ for calcium ions, +Mg+ for magnesium ions, -HS- for humic like substances, -HS-+Ca+-HS- calcium–humic complexes and -HS-+Mg+-HS- magnesium–humic complexes.

Therefore, the strong affinity between divalent cations and NOM substances can also explain the higher (facilitated) transport of Ca^2+^ and Mg^2+^ ions across the dNF40 membrane (i.e., lower retention) at higher NOM concentrations compared to that of Na^+^ ions, whose retention even slightly improved with increasing NOM concentrations. This might be attributed to humic substances, which are known as the major mass fraction of NOM and are negatively charged at pH values of 7–8, due to the dissociation of carboxylic (and phenolic) functional groups, and, as a result, a strong affinity with divalent cations is generated [65]. As the cations pass through the dNF40 membrane, anions must also pass through the membrane to maintain electroneutrality. This resulted in a reduced membrane retention for anions that were already poorly retained by the NF membrane in case of no NOM dosing. Subsequently, the retention of F^−^ ions deteriorated with the increase in the NOM concentration in the feed since membrane fouling increased, but relative to the retention of other monovalent anions (NO_3_^−^ and Cl^−^), F^−^ retention deteriorated less, and only became much worse when the retention of NO_3_^−^ and Cl^−^ reached almost zero, cf. Figure 5. Despite its smaller ionic radius, F^−^ was better retained than Cl^−^ and NO_3_^−^. This can be attributed to its higher charge density that can result in stronger electrostatic repulsion, particularly with the negatively charged membranes. Additionally, the higher dehydration energy of F^−^ can lead to less partial dehydration, further contributing to its enhanced retention [66], and a detailed description of the ionic radius, hydrated radius, and hydration energy of the anions is provided in the Supplementary Data in Section S4 [67,68]. The retention of SO_4_^2−^, on the other hand, was hardly affected by the addition of NOM. It showed a quite limited decrease with the increase in the NOM concentration in the feed. A schematic representation summarizing the previously described effects of NOM addition on the retention of anions and cations in the SMW by dNF40 membranes is presented in Figure 6.

**Figure 7:**
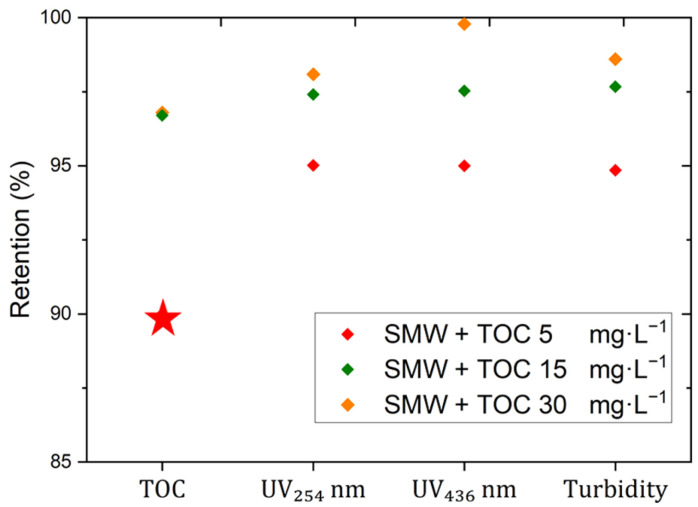
Organic matter retention measured using TOC, UV_254_ nm, VIS_436_ nm, and turbidity during the filtration experiment using SMW containing fluoride with an initial concentration of 20 mg·L^−1^, the addition of different concentrations of TOC of 5, 15, and 30 mg·L^−1^, a crossflow velocity of 0.5 m·s^−1^, a permeate flux of 20 L·m^−2^·h^−1^, and pH 7.5 for 16 h. Red star indicates cases where TOC concentration in the permeate was below the detection limit, with a 90% minimum retention assumed.

Moreover, the retention of NOM substances, measured as TOC and the spectral absorption coefficient of UV_254 nm_ and VIS_436 nm_ at the end of the filtration period of 16 h are presented in Figure 7. It should be noted that for those cases where the TOC concentration in the permeate was below the detection limit, the minimum retention threshold was considered as 90% and represented by a star marker. However, the actual retention could be significantly higher. The results showed an improved retention for NOM substances by dNF40 membranes with increasing NOM concentrations in the feed water. The retention of both the active substances measured by spectral absorption coefficients of UV_254 nm_ and VIS_436 nm_ increased from 95% to >97%. This could be an indication of the progressive formation of a fouling layer on the membrane that affected both F^−^ and NOM retention.

### 3.4. Mini-Plant Filtration Tests Using SMW with NOM at a Constant Concentration Employing Different Crossflow Velocities

**Figure 8:**
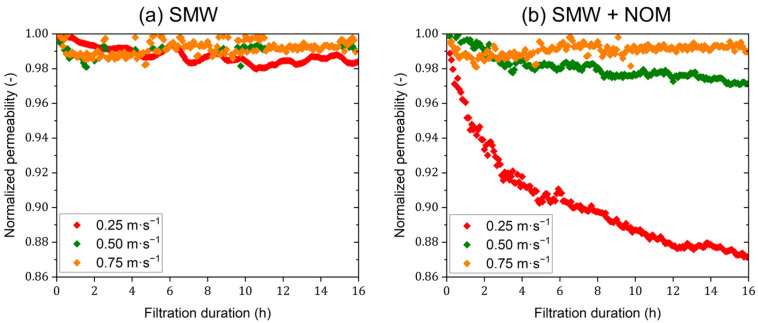
Normalized permeability of (**a**) SMW alone and (**b**) SMW in the presence of NOM at a concentration of 30 mg·L^−1^ TOC using dNF40 with different crossflow velocities of 0.25, 0.50, and 0.75 m·s^−1^, a permeate flux of 20 L·m^−2^·h^−1^, and pH 7.5 for 16 h.

The influences of organic fouling on the separation performance of the dNF40 membrane were additionally investigated by applying three different CFV values (0.25, 0.50, and 0.75 m·s^−1^) during the filtration of SMW without and with an NOM dosage of 30 mg·L^−1^ (as TOC). Figure 8a,b present the normalized permeability curves for both conditions. Regarding filtration experiments using SMW without NOM addition, varying the CFV had no remarkable effects on the normalized permeability declines. In contrast, filtration experiments using SMW with NOM dosing revealed significantly different influences on the fouling behavior of dNF40 membranes for the varying CFV values. The dNF40 membrane exhibited noticeably stable performance with a far-limited normalized permeability decline of 1.5% at a CFV of 0.75 m·s^−1^. A slightly more, but still limited, normalized permeability decline of 3.0% was observed for filtration experiments at a CFV of 0.5 m·s^−1^. Meanwhile, a much more normalized permeability decline of ~13% was found for filtration experiments at a CFV of 0.25 m·s^−1^, indicating that the reduced shear forces at a CFV of 0.25 m·s^−1^ were insufficient to prevent the excessive accumulation of foulants on the membrane surface. These results may suggest that a CFV of 0.5 m·s^−1^ can be considered a threshold value within the currently investigated operating ranges. Above this threshold value, the CFV has a minimal influence on the membrane fouling behavior (as supported by the already very limited normalized permeability decline), while below this value, membrane fouling can increase significantly.

**Figure 9:**
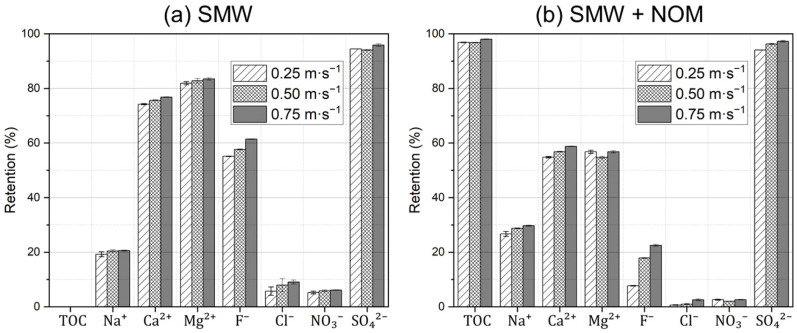
TOC retention and detailed ion retentions measured during the filtration of (**a**) SMW without NOM dosing and (**b**) SMW with NOM dosing with a TOC concentration of 30 mg·L^−1^ using dNF40 with different crossflow velocities of 0.25, 0.50, and 0.75 m·s^−1^, a permeate flux of 20 L·m^−2^·h^−1^, and pH 7.5 for 16 h.

The retention performance for individual ions (and TOC) during the filtration of SMW without and with NOM dosing was also illustrated in Figure 9a,b, respectively. Generally, when comparing the filtration tests with and without NOM dosing, varying the CFV had no major influence on the previously explained mechanistic effects (and retention sequence) in Section 3.3 and Figure 6, i.e., the retention of Ca^2+^ and Mg^2+^ cations decreased whereas the retention of Na^+^ increased. Additionally, the retention of monovalent anions (F^−^, NO_3_^−^, and Cl^−^) decreased, while the retention of SO_4_^2−^ was almost comparable.

However, looking into one series of experiments, one could notice a slight improvement in the retention values for all cations, anions, and TOC with the increase in the CFV. For instance, for filtration experiment series using SMW without NOM dosing, F^−^ ion retention increased from 56% to 59% and 62% with an increase in the CFV from 0.25 m·s^−1^ to 0.50 and 0.75 m·s^−1^, respectively. Similarly, for filtration experiment series using SMW with NOM dosing, F^−^ ion retention increased from 9% to 18% and 23% with an increase in the CFV from 0.25 m·s^−1^ to 0.50 and 0.75 m·s^−1^, respectively. An analogous trend was also reported by Junker et al. [45]. They reported limited influences of varying CFVs on the retention of 600 mg·L^−1^ MgSO_4_; it increased by 3% as the CFV increased from 0.1 m·s^−1^ to 0.6 m·s^−1^ under different permeate flux conditions. However, no improvement in the overall permeability was observed. They concluded that MgSO_4_ retention is governed by the inlet CFV and fiber length, with higher velocities (i.e., >0.6 m·s^−^^1^) mitigating concentration polarization and enhancing retention, while longer fibers promote boundary layer development, reducing retention as recovery increases, as evidenced by a decrease from 90% to 80% when the CFV was reduced from 0.6 to 0.1 m·s^−1^.

### 3.5. Mini-Plant Filtration Tests Using SMW and NOM at a Constant Initial Concentration Employing a Constant Crossflow Velocity at Different Recoveries

**Figure 10:**
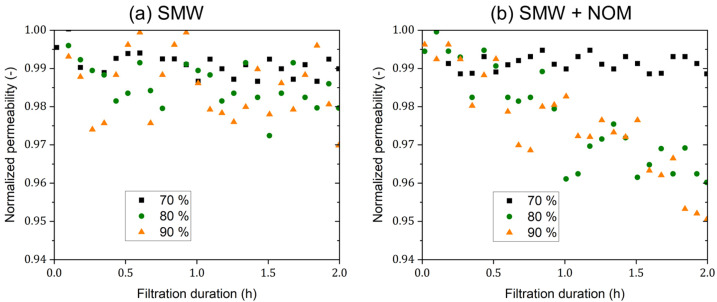
Normalized permeability curves for filtration experiments using (**a**) SMW without NOM dosing and (**b**) SMW with NOM dosing with a TOC concentration of 5 mg·L^−1^ using dNF40 at different final recovery values of 70, 80, and 90%, a permeate flux of 20 L·m^−2^·h^−1^, crossflow velocity of 0.5 m·s^−1^, and pH 7.5 for 2 h.

These experiments were conducted to simulate different fouling scenarios at filtration conditions close to the full-scale application conditions, where the concentrations of both ions and organics may increase during the filtration process, as well as fouling exacerbation on the membrane surface. The normalized permeability curves for batch filtration experiments using SMW without and with NOM dosing are presented in Figure 10a,b, respectively. Generally, the normalized permeability decline for all filtration experiments was <5%, indicating a very good overall membrane performance under the employed conditions. Concerning the filtration experiments using SMW without NOM dosing, the differences in normalized permeability trends upon operation at different recovery rates were limited; values were in range of 1.0–2.5%. While in the case of filtration experiments using SMW with a NOM concentration of 5 mg·L^−1^ (as TOC), more different normalized permeability trends were observed because of the different fouling extents; values of 1.0%, 4.0%, and 5.0% were calculated for the batch operation at recovery rates of 70, 80, and 90%, respectively.

**Figure 11:**
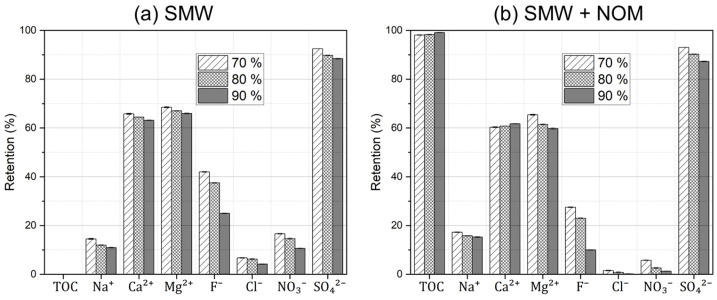
TOC retention and detailed ion retentions measured during the filtration of (**a**) SMW without NOM dosing and (**b**) SMW with NOM dosing at a TOC concentration of 5 mg·L^−1^ using dNF40 at different final recovery values of 70, 80, and 90%, a permeate flux of 20 L·m^−2^·h^−1^, and pH 7.5 for 2 h.

Furthermore, the separation performance of dNF40 membranes is presented in Figure 11. In contrast to filtration experiments under steady-state conditions, the concentrations of cations, anions, and NOM substances were increasing with time (i.e., filtration progression) up to certain final values depending on their concentration factor, which were different depending on the recovery rate and the respective retention. For instance, in case of filtration experiments using SMW without NOM addition, the increasing salt concentration in the feed–retentate channel led to a systematic reduction in the membrane retention of all anions and cations. This reduction was significantly higher for monovalent anions (F^−^, NO_3_^−^, and Cl^−^) than the divalent SO_4_^2−^ ions. These anions are smaller and less charged; hence they can pass through the membrane to a higher extent as more cations pass through the membrane (see Figure 11a). Such an overall reduction in salt retention at high recovery rates should be related to the increased, already complex concentration polarization. For the filtration experiments using SMW with NOM dosing, the mechanistic effects of the NOM fouling layer on the membrane retention of all cations and anions (described in Section 3.3.) remained generally applicable. However, when comparing with Figure 5, one could see different retention trends for Ca^2+^ and Na^+^, in spite of the overall reduced retention values in Figure 11b. The Ca^2+^ retention was found to slightly improve at higher recovery rates, whereas it exhibited a majorly decreasing trend in steady-state experiments. Despite the complex water matrix nature, these results might be attributed to a kind of cake-reduced concentration polarization effect, where more Ca^2+^ ions were retained (via NOM-Ca^2+^ complexation) inside the exacerbated fouling layer on the membrane surface at high recovery values. Simultaneously, the transport of Na^+^ ions through the membrane into the permeate was promoted to maintain electroneutrality; hence, this resulted in reduced Na^+^ retention, as shown in Figure 11b.

## 4. Conclusions and Application Remarks

This work explored the applicability of the commercially available 400 kDa poly-electrolyte multilayer-based hollow-fiber nanofiltration membrane dNF40 for fluoride removal from contaminated water sources with high TDS and NOM contents under varying operating conditions. The removal of fluoride could potentially be an additional benefit if the NF membrane is used to reduce the NOM and/or TDS concentrations. The influences of organic fouling, feed salinity, and different operating conditions on the separation efficiency of dNF40 membranes were studied. The following findings can be outlined:

- dNF40 exhibited an F^−^ retention of >70% during single salt retention experiments (without NOM and competitive ions), which increased with higher membrane flux but decreased with an increasing initial feed concentration.
- For SMW with a high TDS as the feed water, F^−^ retention decreased due to ion competition, reaching values as low as 25% under the most challenging conditions (high recovery and a low CFV).
- For SMW with high TDS and NOM contents as the feed water, F^−^ retention further decreased down to about 10% under the most challenging conditions (high recovery, a low CFV, and extended filtration duration) due to fouling effects. Facilitated transport of the divalent cations Ca^2+^ and Mg^2+^ could be observed as they accumulated in the organic fouling layer. While SO_4_^2−^ retention remained relatively stable, the retention of monovalent anions (NO_3_^−^, Cl^−^, and F^−^) decreased dramatically due to drag effects. On the other hand, Na^+^ retention improved slightly to maintain electroneutrality.
- Increasing the crossflow velocity resulted in a slightly improved separation performance for NOM and all ions. Meanwhile, a crossflow velocity of 0.5 m·s^−1^ was identified as the threshold value to maintain consistent performance of dNF40 membranes in this study; this also matches the manufacturer’s recommendation.
- The complex interactions between ions of varying charges and sizes, organic substances, and membrane system operating conditions create simultaneous and competing effects, making precise predictions of the retention of individual anions and cations challenging. As a result, only general trends can be identified.
- It is important to consider that the separation performance of polyelectrolyte multilayer membranes decreases with increasing feed salinity due to structural changes in the multilayers, although these changes remain reversible.

In summary, the dNF40 membrane offers potential for fluoride removal, but its effectiveness is significantly influenced by the operating conditions, feed composition, and fouling effects, highlighting the need for a careful consideration of these factors in practical applications. While not ideal for highly contaminated water requiring extensive fluoride removal, it is well-suited for applications prioritizing selective sulfate, NOM and micropollutant removal, such as advanced wastewater treatment, while allowing salt passage [32].

The ability of dNF40 to maintain high separation performance with minimal or no pretreatment may reduce the operational costs and improve overall water treatment efficiency. Compared to brackish water reverse osmosis membranes (e.g., BW30) and NF90, dNF40 membranes pose a low risk of scaling, potentially allowing for antiscalant-free desalination processes, even at higher recovery rates, because of their selective ion separation capabilities, and they may offer advantages in brine management, given the relatively low concentration factors at typical inland recovery rates.

## Figures and Tables

**Table 1:** Ion composition and physicochemical characteristics of the SMW solutions employed in this study.

Samples	TOC (mg·L^−1^)	UV_254_ (cm^−1^)	VIS_436_ (cm^−1^)	Turbidity (FNU)	Conductivity (μS·cm^−1^)	Ion Concentration (mg·L^−1^)						
						Na^+^	Ca^2+^	Mg^2+^	F^−^	Cl^−^	NO_3_^−^	SO_4_^2−^
* PS	30 ± 0.30	1.2 ± 0.02	1.08 ± 0.01	20.6 ± 0.40	170 ± 1.30	32	2	2	<DL	26	1	38
** Real					8721 ± 1.30	1400	325	234	2.5	1840	11	1830
SMW					8960 ± 1.30	1393	330	235	20	2004	50	1827
SMW + NOM (5 mg·L^−1^)	5 ± 0.30	0.2 ± 0.02	0.03 ± 0.01	2.9 ± 0.40	8992 ± 1.30	1404	330	235	20	2003	52	1850
SMW + NOM (15 mg·L^−1^)	15 ± 0.30	0.7 ± 0.02	0.12 ± 0.01	9.4 ± 0.40	9020 ± 1.30	1420	330	234	20	2010	50	1862
SMW + NOM (30 mg·L^−1^)	30 ± 0.30	1.2 ± 0.02	1.08 ± 0.01	21.8 ± 0.40	9130 ± 1.30	1435	332	236	20	2225	51	1892

* A representative composition of reference potting soil with TOC 30 mg·L^−1^; the results indicated that the F^−^ concentration of the reference potting soil was below the detection limit (<DL) of 0.6 mg·L^−1^. ** Real water collected from the Gafsa region. All TOC, spectral absorption coefficients measured at two wavelengths of UV_254 nm_ and VIS_436 nm_, turbidity and conductivity measurements were repeated at least 3 times; standard deviation values were calculated.

## Data Availability

The raw data supporting the conclusions of this article will be made available by the authors on request.

## Supplementary Materials

The following supporting information can be downloaded at https://www.mdpi.com/article/10.3390/membranes15040110/s1. Figure S1. Schematic diagram for the SMW preparation utilized in the filtration experiments; Figure S2. TOC retention and detailed ion retentions measured during SMW filtration experiment containing fluoride with initial concentration of 20 mg·L^−1^ alone or with addition of different concentrations of TOC 5, 15 and 30 mg·L^−1^, crossflow velocity of 0.5 m·s^−1^, permeate flux of 20 L·m^−2^·h^−1^ and pH 7.5, permeate samples were collected at different filtration durations of 2, 4, 8 and 16 h; Table S1. A quantitative analysis of the cleaning efficiency obtained during different filtration experiments; Table S2. Ionic radius, hydrated ionic radius and hydration-free energy for studied ions during filtration experiments.
