# Supplementary material for: Impacts of Natural Organic Matter and Dissolved Solids on Fluoride Retention of Polyelectrolyte Multilayer-Based Hollow Fiber Nanofiltration Membranes
Source: Membranes (Basel). 2025 Apr 2;15(4):110. doi: 10.3390/membranes15040110 (PMC12029039; doi:10.3390/membranes15040110)
Supplement: Supplementary file 1 [file membranes-15-00110-s001.zip › membranes-3480749-supplementary.pdf]

Supporting Information

Impacts of Natural Organic Matter and Dissolved Solids on Fluoride Retention of Polyelectrolyte Multilayer-Based Hollow Fiber Nanofiltration Membranes

Hussein Abuelgasim [1,†], Nada Nasri [2,†], Martin Futterlieb [1], Radhia Souissi [2,3], Fouad Souissi [2,3], Stefan Panglisch [1,4,5,*] and Ibrahim M. A. ElSherbiny [1,*]

[1] Chair for Mechanical Process Engineering & Water Technology, University of Duisburg-Essen, Lotharstraße 1, 47057 Duisburg, Germany; 7ussien3861@gmail.com (H.A.); martin.futterlieb@uni-due.de (M.F.)
[2] Laboratoire des Matériaux Utiles, Institut National de Recherche et d'Analyse Physico-Chimique (INRAP), Technopark of Sidi Thabet, Ariana 2020, Tunisia; nada.nasri66@gmail.com (N.N.); radhia.souissi@inrap.rnrt.tn (R.S.); foued.souissi@fst.utm.tn (F.S.)
[3] Department of Geology, Faculty of Sciences of Tunis, University of Tunis El Manar, Tunis 2092, Tunisia
[4] DGMT German Society of Membrane Technology, Geschäftsstelle ZWU, Universitätsstraße 2, 45141 Essen, Germany
[5] IWW Water Centre, Moritzstraße 26, 45476 Mülheim an der Ruhr, Germany
* Correspondence: stefan.panglisch@uni-due.de (S.P.); ibrahim.elsherbiny@uni-due.de (I.M.A.E.)
† These two authors contributed equally to this work.

S1. Schematic diagram for the SMW preparation utilized in the filtration experiments:

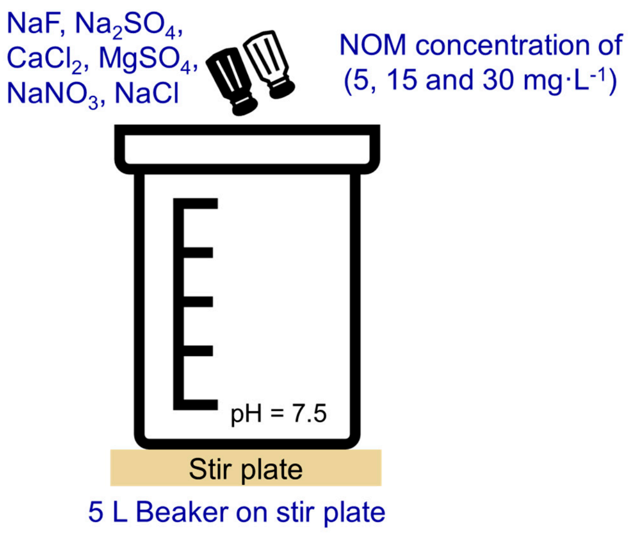

Salt	Concentration / (mg·L^{-1})
NaF	44
NaCl	2340
$NaNO_3$	70
Na_2SO_4	1330
$CaCl_2$	916
$MgSO_4$	1164

Figure S1. Schematic diagram for the SMW preparation utilized in the filtration experiments

S2. Quantitative analysis of the cleaning efficiency calculated in terms of permeability recovery rate during different filtration experiments:

The permeability recovery rate after chemical cleaning step was determined using Equation (S1).

$$\text{Equation (S1): Permeability recovery rate (\%)} = \frac{W_r}{W_i} \text{ x } 100$$

where W_r is the membrane permeability after the chemical cleaning step ($L \cdot m^{-2} \cdot h^{-1} \cdot bar^{-1}$), W_i is the initial membrane permeability expressed as pure water permeability ($L \cdot m^{-2} \cdot h^{-1} \cdot bar^{-1}$), which was determined by 5.4 in this study.

Table S1. A quantitative analysis of the cleaning efficiency obtained during different filtration experiments

Samples	Filtration condition	Permeability after cleaning	Permeability recovery rate / (%)
NaF (77 $mg \cdot L^{-1}$)	30 $L \cdot m^{-2} \cdot h^{-1}$, 0.5 $m \cdot s^{-1}$ & 16 h	5.40 ± 0.01	99.9 ± 0.09
Glucose (200 $mg \cdot L^{-1}$)	13 $L \cdot m^{-2} \cdot h^{-1}$, 0.5 $m \cdot s^{-1}$ & 2 h	5.40 ± 0.01	99.9 ± 0.09
Glucose + NaCl (600 $mg \cdot L^{-1}$)	13 $L \cdot m^{-2} \cdot h^{-1}$, 0.5 $m \cdot s^{-1}$ & 2 h	5.40 ± 0.01	99.9 ± 0.09
Glucose + NaCl (2,000 $mg \cdot L^{-1}$)	13 $L \cdot m^{-2} \cdot h^{-1}$, 0.5 $m \cdot s^{-1}$ & 2 h	5.38 ± 0.01	99.6 ± 0.19
Glucose + NaCl (4,000 $mg \cdot L^{-1}$)	13 $L \cdot m^{-2} \cdot h^{-1}$, 0.5 $m \cdot s^{-1}$ & 2 h	5.37 ± 0.02	99.4 ± 0.28
Glucose + NaCl (10,000 $mg \cdot L^{-1}$)	13 $L \cdot m^{-2} \cdot h^{-1}$, 0.5 $m \cdot s^{-1}$ & 2 h	5.36 ± 0.01	99.2 ± 0.09
Glucose + NaCl (29,200 $mg \cdot L^{-1}$)	13 $L \cdot m^{-2} \cdot h^{-1}$, 0.5 $m \cdot s^{-1}$ & 2 h	5.33 ± 0.01	98.6 ± 0.09
SMW	20 $L \cdot m^{-2} \cdot h^{-1}$, 0.5 $m \cdot s^{-1}$ & 16 h	5.38 ± 0.01	99.6 ± 0.09
SMW + NOM (5 $mg \cdot L^{-1}$)	20 $L \cdot m^{-2} \cdot h^{-1}$, 0.5 $m \cdot s^{-1}$ & 16 h	5.35 ± 0.01	99.0 ± 0.09
SMW + NOM (15 $mg \cdot L^{-1}$)	20 $L \cdot m^{-2} \cdot h^{-1}$, 0.5 $m \cdot s^{-1}$ & 16 h	5.32 ± 0.01	98.4 ± 0.09
SMW + NOM (30 $mg \cdot L^{-1}$)	20 $L \cdot m^{-2} \cdot h^{-1}$, 0.5 $m \cdot s^{-1}$ & 16 h	5.30 ± 0.01	98.1 ± 0.09
SMW	20 $L \cdot m^{-2} \cdot h^{-1}$, 0.25 $m \cdot s^{-1}$ & 16 h	5.38 ± 0.01	99.4 ± 0.09
SMW	20 $L \cdot m^{-2} \cdot h^{-1}$, 0.50 $m \cdot s^{-1}$ & 16 h	5.38 ± 0.01	99.4 ± 0.09
SMW	20 $L \cdot m^{-2} \cdot h^{-1}$, 0.75 $m \cdot s^{-1}$ & 16 h	5.38 ± 0.01	99.4 ± 0.09
SMW + NOM (30 $mg \cdot L^{-1}$)	20 $L \cdot m^{-2} \cdot h^{-1}$, 0.25 $m \cdot s^{-1}$ & 16 h	5.22 ± 0.01	96.6 ± 0.09
SMW + NOM (30 $mg \cdot L^{-1}$)	20 $L \cdot m^{-2} \cdot h^{-1}$, 0.50 $m \cdot s^{-1}$ & 16 h	5.29 ± 0.01	98.0 ± 0.19
SMW + NOM (30 $mg \cdot L^{-1}$)	20 $L \cdot m^{-2} \cdot h^{-1}$, 0.75 $m \cdot s^{-1}$ & 16 h	5.35 ± 0.01	99.0 ± 0.19
SMW	20 $L \cdot m^{-2} \cdot h^{-1}$, 0.5 $m \cdot s^{-1}$, 70 % & 2 h	5.35 ± 0.01	99.0 ± 0.19
SMW	20 $L \cdot m^{-2} \cdot h^{-1}$, 0.5 $m \cdot s^{-1}$, 80 % & 2 h	5.32 ± 0.01	98.4 ± 0.09
SMW	20 $L \cdot m^{-2} \cdot h^{-1}$, 0.5 $m \cdot s^{-1}$, 90 % & 2 h	5.30 ± 0.01	98.1 ± 0.09
SMW + NOM (5 $mg \cdot L^{-1}$)	20 $L \cdot m^{-2} \cdot h^{-1}$, 0.5 $m \cdot s^{-1}$, 70 % & 2 h	5.30 ± 0.01	98.1 ± 0.09
SMW + NOM (5 $mg \cdot L^{-1}$)	20 $L \cdot m^{-2} \cdot h^{-1}$, 0.5 $m \cdot s^{-1}$, 80 % & 2 h	5.25 ± 0.01	97.1 ± 0.09
SMW + NOM (5 $mg \cdot L^{-1}$)	20 $L \cdot m^{-2} \cdot h^{-1}$, 0.5 $m \cdot s^{-1}$, 90 % & 2 h	5.25 ± 0.01	96.2 ± 0.09

<u>S3. A detailed description of respective retention values observed during different filtration durations:</u>

TOC retention and detailed ion retentions measured during SMW filtration experiment containing fluoride with initial concentration of 20 mg·L^{-1} alone or with addition of different concentrations of TOC 5, 15 and 30 mg·L^{-1}, CFV of 0.5 m·s^{-1}, permeate flux of 20 L·m^{-2}·h^{-1} and pH 7.5 for 16 h, and permeate samples were collected at different filtration durations of 2, 4, 8 and 16 h with the specific time scale to address the time-resolved retention rate change.

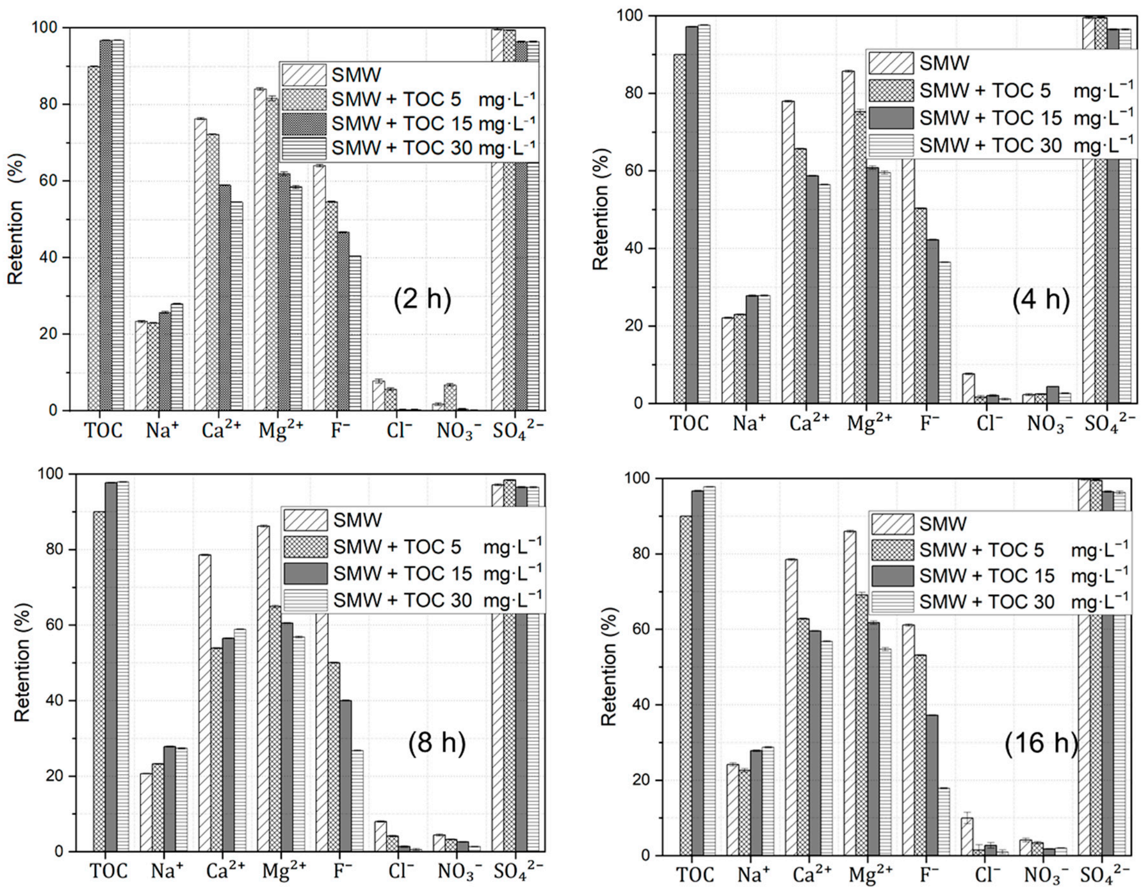

Figure S2. TOC retention and detailed ion retentions measured during SMW filtration experiment containing fluoride with initial concentration of 20 mg·L^{-1} alone or with addition of different concentrations of TOC 5, 15 and 30 mg·L^{-1}, crossflow velocity of 0.5 m·s^{-1}, permeate flux of 20 L·m^{-2}·h^{-1} and pH 7.5, permeate samples were collected at different filtration durations of 2, 4, 8 and 16 h

S4. A detailed description of ionic radius, hydrated ionic radius, and hydration-free energy for studied ions within the scope of the study:

The variation in ion selectivity and retention can be attributed to differences in ion hydration. Ion retention follows a trend corresponding to their respective hydration energies, with highly hydrated ions exhibiting lower membrane permeability due to increased resistance to transport.

Table S2. Ionic radius, hydrated ionic radius and hydration-free energy for studied ions during filtration experiments

Ion	Ionic radius / (nm)[a]	Hydrated ionic radius / (nm)[a]	Hydration-free energy / ($kJ \cdot mol^{-1}$)[b]
Na^{+}	0.117	0.358	-365
Ca^{2+}	0.100	0.412	-1505
Mg^{2+}	0.072	0.428	-1830
F^{-}	0.116	0.352	-465
Cl^{-}	0.164	0.332	-340
NO_3^{-}	0.179	0.340	-300
SO_4^{2-}	0.240	0.380	-1080

[a] The ionic radius and hydrated radius of the ions were obtained from Volkov et al. (see Reference [67] in the main manuscript).

[b] The hydration-free energy values were obtained from Marcus et al. (see Reference [68] in the main manuscript).
